# Economic valuation of a mesocarnivore’s impact management

**DOI:** 10.1007/s11356-024-33398-4

**Published:** 2024-04-22

**Authors:** Vasileios J. Kontsiotis, Foteini Emmanouilidou, Vasilios Liordos

**Affiliations:** Present Address: Department of Forest and Natural Environment Sciences, International Hellenic University, P.O. Box 172, 66100 Drama, Greece

**Keywords:** Human-wildlife conflict, Red fox, Contingent valuation, Willingness to pay, Impact management, Cognitions, Emotions, Sociodemographics

## Abstract

Red foxes (*Vulpes vulpes*) have interacted with humans during their common history. We used a contingent valuation method to assess the economic value of the management of this mesocarnivore’s negative impacts. We carried out face-to-face interviews with 746 Greek residents, using a multiple-bounded discrete choice approach to estimate willingness to pay (WTP) for red fox management under three impact situations: attack domestic animals, reduce game, carry disease. About 51.9%, 33.0%, and 81.1% of the respondents stated a mean WTP of €34.1, €44.9, and €72.1 for each situation, respectively. The total annual amounts of €18.7 million, €15.7 million, and €61.7 million could be collected from the target population for red fox management when they attack domestic animals, reduce game, and carry disease, respectively. Attitudes and likeability toward foxes were negatively associated while knowledge about foxes and fear of them were positively associated with WTP. The younger, richer, less educated, rural, farmers, hunters, and pet owners were generally more willing to pay for red fox management across situations. Females were more willing to pay for managing predation on domestic animals, while males were more willing to pay for managing predation on game. Our findings showed that the Greek residents highly value the management of red foxes in all impact situations and would be valuable for further advising the management process.

**Supplementary Information:**

The online version contains supplementary material available at 10.1007/s11356-024-33398-4.

## Introduction

People and wildlife have interacted and competed for resources since the dawn of humanity (Nyhus 2016). Nowadays, the increase in the human population and the levels of urbanization increase the possibility of human-wildlife interactions that often result in conflicts. The International Union for the Conservation of Nature Species Survival Commission Human-Wildlife Conflict Task Force defined human-wildlife conflict as *struggles that emerge when the presence or behavior of wildlife poses actual or perceived, direct, and recurring threat to human interests or needs, leading to disagreements between groups of people and negative impacts on people and/or wildlife* (IUCN SSC HWCTF 2020). Negative wildlife impacts include damage to crops, predation on livestock and pets, collisions with vehicles, damage to property, and attacks on humans resulting in injury or death (Manfredo 2008).

Wildlife managers aim to reduce wildlife impacts by selecting and implementing the most suitable management strategy for each situation (Teel and Manfredo 2010). However, impact management cannot be successful without the consensus of society (Manfredo 2008). Therefore, many studies have examined the acceptability of strategies for managing wildlife impacts in different situations (e.g., Frank et al. 2015; Sponarski et al. 2015; Shuttlewood et al. 2016; Doney et al. 2018; Liordos et al. 2019; Kontsiotis et al. 2020; Martínez-Jauregui et al. 2020). Although such attitudinal approaches are necessary and valuable, they do not allow for the estimation of public funds that could be collected for implementing wildlife impact management programs. The preferences for wildlife management strategies are goods with non-use values, meaning that they cannot be commercially traded and their value cannot be directly measured (Perman et al. 2003; Greene 2018) but can be indirectly evaluated using stated preferences methods (Jones-Walters and Mulder 2009; Morse-Jones et al. 2012). The contingent valuation method CVM) is the most frequently applied stated preference method, that assigns value to non-use goods by creating a hypothetical market where consumers can express their preferences for the amenities in question in monetary terms (Perman et al. 2003; Greene 2018). By using the CVM in the wildlife domain, people can assign an economic value to species-specific wildlife impact management programs through willingness to pay (WTP) surveys. Studies on WTP for wildlife damage have been conducted on species such as brush-tailed possums (*Trichosurus vulpecula*) (Kerr and Cullen 1995), red deer (*Cervus elaphus*) (Bowker et al. 2003; Martínez-Jauregui et al. 2020), African elephants (*Loxodonta africana*) (van Eden et al. 2016), Asian elephants (*Elephas maximus*) (Neupane et al. 2017), African elephants, African buffalos (*Syncerus caffer*), African lions (*Panthera leo*), and spotted hyenas (*Crocuta crocuta*) (Mhuriro-Mashapa et al. 2017), and wild boar (*Sus scrofa*) (Ellis et al. 2021). These studies have shown that WTP surveys not only can allow for assessing management preferences, but they can also allow for the economic valuation, a proxy of the social value, of wildlife impact management (Perman et al. 2003).

The red fox (*Vulpes vulpes*), hereafter fox, the most widely distributed among the carnivore species, is found in the northern hemisphere and has also been introduced to Australia and New Zealand (Hoffmann and Sillero-Zubiri 2021). Foxes are both habitat and food generalists. While they prefer a mixture of scrub, woodland, and farmland, they also flourish in human-modified landscapes, such as agricultural land (Harris and Lloyd 1991). Omnivorous animals as they are, they mostly take rodents in Europe, but will eat anything, from fruits and berries to invertebrates, birds, rabbits, hares, lambs, sheep, poultry, and to carrion and food remains (Papageorgiou et al. 1988; Goszcynski and Wasilewski 1992; Serafini and Lovari 1993; O'Mahony et al. 1999; Vlachos et al. 2006; Díaz-Ruiz et al. 2013; Pagh et al. 2015). Foxes have lived in close association with humans for ages, are considered as beautiful and attractive, and also as useful animals in controlling the rodent population (König 2008). On the other hand, foxes can also be harmful to people and other animals as they are vectors of zoonotic diseases (Tasioudi et al. 2014) and also because they prey upon domestic animals such as poultry and lamb (Papageorgiou et al. 1988; White et al. 2000; Moberly et al. 2003) or game animals such as European wild rabbit (*Oryctolagus cuniculus*), hares (*Lepus* spp.), and partridges (*Alectoris* spp. and grey partridge *Perdix perdix*) (Papageorgiou et al. 1988; Reynolds and Tapper 1996; Vlachos et al. 2006; Reynolds et al. 2010).

Farmers often complain about losses of farm animals to foxes, while hunters complain that foxes reduce game populations (Reynolds and Tapper 1996; Tapper et al. 2010). However, losses are usually low for indoor-farmed animals although the predation can sometimes be high, especially for outdoor-farmed animals (White et al. 2000; Moberly et al. 2003). Papageorgiou et al. (1988) found that small rodents were the main prey of foxes in Greece, followed by lambs and piglets. In another study from Greece, Vlachos et al. (2006) found a prevalence of brown hares (*Lepus europaeus*) in fox diet. Although microtine rodents are the favorite prey of foxes in Europe, research has shown that these canids will switch to alternative prey, including locally abundant game populations, when their main prey is scarce (Goszcynski and Wasilewski 1992; Knauer et al. 2010; Díaz-Ruiz et al. 2013). Fencing of property and hunting as a game have been used to protect farm animals and game populations from fox predation in Greece, respectively (Liordos et al. 2020a).

Foxes are the main reservoirs and vectors of the deadly rabies virus in Europe which causes each year about 55,000 deaths of people worldwide (Tasioudi et al. 2014). Fox-mediated rabies has been eliminated from most Western and Central European countries due to the implementation of oral vaccination programs for foxes (Freuling et al. 2013), although the disease still occurs in other European countries, with 4,884 cases in animals recorded in 2012 (Tasioudi et al. 2014). Greece was considered rabies-free since 1987 with no human cases since 1970 (Tasioudi et al. 2014). However, the disease reemerged in 2012–2013, with 17 animals diagnosed with rabies, including 14 foxes, two shepherd dogs, and one cat (Tsiodras et al. 2013). Subsequently, 104 people were exposed to rabid animals and received prophylactic treatment (Tsiodras et al. 2013). Rabid foxes probably crossed the borders with Turkey, Northern Macedonia, and Bulgaria, where rabies is reported in wild and domestic animals (Picard-Meyer et al. 2013). Oral vaccination baits and collection of individuals for disease inspection are the management strategies that have been used in Greece for rabies elimination (Tasioudi et al. 2014).

Liordos et al. (2020a) examined the preferences of northern Greek residents for managing fox impacts in the three most common impact situations, namely when foxes attack domestic animals, reduce game, and transfer rabies. They found that people preferred non-lethal management strategies over lethal strategies. But taking no action was relatively unacceptable and lethal control was more acceptable with increasing severity of the conflict, i.e., livestock and game predation versus rabies transmission. The use of the CVM method could further such research because not only it could help determine public support for fox impact management (i.e., manage or do not manage a situation), but it would also allow for identifying potential funding sources for managing foxes under different impact situations. There are two formats for WTP questions, the continuous (e.g., open-ended and payment card) and discrete choice formats (Greene 2018). Discrete choice formats can be single-bounded (the respondent has to select between two options: to pay a certain amount for the good or service or not to pay at all), double-bounded (the respondent has to select between two different payment amounts: the initial bid and a higher or lower than the initial bid), or multiple-bounded (the respondent is asked to express payment certainty on a large number of amounts). We used the multiple-bounded discrete choice CVM because it provides respondents with a limited set of options, thus reducing bias in answers compared to continuous formats (Brown et al. 1996) and also allows for avoiding the problems associated with the choice of payment amounts required to implement either a single-bounded or double-bounded model (Welsh and Poe 1998).

Although everyone in general could be impacted by foxes as they carry diseases, it is mostly farmers (when livestock are attacked) and hunters (when game species are killed) who are mostly affected by foxes (Harris and Lloyd 1991; Reynolds and Tapper 1996; Tapper et al. 2010). Previous studies have shown that farmers are more willing to accept and pay for impact management than non-farmers when wildlife attack their livestock or damage their crops (Liordos et al. 2017b; Mhuriro-Mashapa et al. 2017; van Eeden et al. 2021). Furthermore, hunters are more willing to accept impact management than non-hunters when wildlife reduce their favorite game (Frank et al. 2015; Kontsiotis et al. 2020; Liordos et al. 2017b, 2020a). The public, especially those with a higher probability of being exposed to wildlife (i.e., rural residents), are more willing to accept the management of life-threatening situations, such as disease transmission, than managing less severe situations, such as crop, livestock, or game damage (Frank et al. 2015; Kontsiotis et al. 2020; Liordos et al. 2017b, 2020a).

Jacobs et al. (2012) showed that apart from cognitions, emotions also explain a large part of the variation in models for explaining wildlife conservation and management preferences and perceptions. They recommended the inclusion of emotions, along with cognitions, in studies in the human dimensions of wildlife domain that will help to increase the understanding of our relationships and interactions with wildlife. Arnold (1960) defined emotion as *the felt tendency toward anything intuitively appraised as good (beneficial), or away from anything intuitively appraised as bad (harmful). This attraction or aversion is accompanied by a pattern of physiological changes organized toward approach or withdrawal. The pattern differs for different emotions* (p. 182). Likeability and fear are among the most studied emotions of humans toward wildlife (Tisdell et al. 2006; Jacobs et al. 2014a, b). Studies have reported higher support and WTP for conservation and lower support and WTP for impact management of more likeable and less fearsome animals compared to less likeable and more fearsome animals (e.g., Knight 2008; Johansson et al. 2012; Liordos et al. 2017a).

High factual knowledge about the biology and behavior of wildlife species usually corresponds to positive attitudes, high support, and high WTP for their conservation (Parker et al. 2014; Liordos et al. 2019). Furthermore, it has been shown that with increasing factual knowledge about the biology and behavior of Eurasian otters (*Lutra lutra*), the acceptability of doing nothing increased, while the acceptability of invasive strategies (both lethal and non-lethal) decreased (Liordos et al. 2019). When sociodemographic characteristics are concerned, age, gender, income, level of education, place of residence, and pet ownership are among the best predictors of willingness to support wildlife species conservation and management. Young, rich, urban residents, females, people with higher education, and pet owners are usually more supportive of wildlife conservation and less supportive of wildlife impact management, especially in less severe situations and for more invasive management strategies (e.g., Teel and Manfredo 2010; Smith et al. 2014; Frank et al. 2015; Shuttlewood et al. 2016; Liordos et al. 2020b).

Our main objective was to estimate the WTP of Greek residents for managing foxes in three different conflict situations: 1) when foxes attack domestic animals, 2) when foxes reduce game populations, and 3) when foxes carry disease. Our second objective was to predict the effects of attitudes, knowledge, emotions (likeability, fear), and sociodemographics (age, gender, income, level of education, place of residence, occupation, hunting membership, pet ownership, participation in outdoor recreation) on WTP.

## Materials and methods

### Sample collection

The study was carried out between March and May 2017 in north Greece, in the regions of Western Macedonia, Central Macedonia, and Eastern Macedonia and Thrace (40^ο^06΄04΄΄Ν, 20^ο^40΄56΄΄E – 41^ο^06΄15΄΄Ν, 26^ο^19΄42΄΄E), an area with a population of roughly 2.77 million people and 1.06 million households (ELSTAT 2011). Data was collected using an on-site respondent-completed survey (Vaske 2019). Before conducting the official survey, we tested the questionnaire with 30 random people to assess its clarity, completeness, and the time taken for completion. We visited most neighborhoods covering areas of diverse socioeconomic status, in cities, towns, and villages in the study area. These visits took place during open market hours (09:00 h -15:00 h and 17:00 h—21:00 h, from Monday to Saturday to survey a large and diverse sample of people. To avoid surveying people from the same household, each neighborhood was visited only once and when people were encountered in groups, we asked them if they belonged to the same household. The survey was conducted by one researcher (F.E.) to avoid inter-researcher bias. F.E. asked every fifth person she encountered to participate in the survey by reading and responding to questions in the questionnaire, with the help of the researcher when needed (respondent-completed survey; Vaske 2019). If more than five persons had passed in front of the researcher at the time when the questionnaire was completed by a respondent, the researcher requested the next first person she encountered to participate in the survey. F.E. also visited farmers' cooperatives and hunting clubs to increase the participation of farmers and hunters in the survey. Respondents needed about 40 min to complete the questionnaire.

### Questionnaire development

The questionnaire consisted of three parts (Table S1). In the first part, we asked the respondents about their willingness to pay for managing red foxes under three impact situations: (1) foxes attack domestic animals (e.g., lambs, poultry, pets), (2) foxes reduce game species populations, such as brown hare and grey partridge, and (3) foxes carry deadly diseases transferrable to people and animals (e.g., rabies). We invoked the respondents’ attention here to treat each situation as independent of the others, meaning that they would be asked to pay for one program only. WTP was assessed in two steps. In the first step, the respondents were asked, with situation 1 as an example “When there is evidence that foxes attack domestic animals, such as lambs, poultry, and pets, causing considerable economic damage, would you support a governmental management program to reduce fox impact by paying an amount annually for the next five years?” Possible answers were ‘‘yes’’ and ‘‘no.’’ The second step included only the respondents that replied yes in the first step: ‘‘Below are listed several amounts of an annual tax that you will have to pay for the next five years for reducing fox impact. Check for each amount how certain you are about paying that amount.’’ The amounts were €1, €5, €10, €20, €40, €80, €150, €300, and €500, and possible answers were ‘‘definitely yes,’’ ‘‘probably yes,’’ ‘‘not sure,’’ ‘‘probably no’’ or ‘‘definitely no’’. These amounts were selected to capture the socioeconomic status in Greece (ELSTAT 2011), also following proposals by Johansson et al. (2012) and Broberg and Brännlund (2008).

In the second part, we measured the attitudes toward and knowledge about foxes (Table S1). We asked respondents to rate each of five attitude and four knowledge statements on a 5-point scale as: “strongly disagree” (1), “disagree” (2), “neither” (3), “agree” (4); or “strongly agree” (5). We also measured the degree of the respondents’ likeability and fear of foxes. We asked the respondents: (a) how safe or afraid would they feel if encountering foxes (“very safe” (1), “safe” (2), “neither” (3), “afraid” (4); or “very afraid” (5)), and (b) how strongly they like or dislike foxes (“strongly dislike” (1), “dislike” (2), “neither” (3), “like” (4); or “strongly like” (5)).

In the third part, we asked survey respondents about sociodemographic characteristics (Table S1): (1) gender (female or male), (2) age (in years), 3) education (recorded as higher or lower), (4) annual household income (€), (5) current residence (recorded as rural: villages and towns < 10,000 inhabitants, or urban: towns and cities > 10,000 inhabitants), (6) occupation (recorded as farmer or non-farmer), (7) hunting membership (recorded as hunter or non-hunter), and (8) pet ownership (recorded as pet owner or non-pet owner).

### The econometric model

A random utility econometric model was used, including two steps. A binary logistic model on the probability of paying for the implementation of a fox impact management program was firstly performed (Greene 2018), with the WTP (yes = 1, no = 0) as the dependent variable and attitude, knowledge, likeability, fear, and the sociodemographic variables of Table 1 as the independent variables.

**Table 1:** Variables used in the willingness to pay (WTP) for red fox management analysis

Variable	Definition	Mean	SD	Min	Max
WTP	1 if the participant is willing to pay for the management of red foxes in Greece, when they attack domestic animals	0.519	0.5	0	1
WTP	1 if the participant is willing to pay for the management of red foxes in Greece, when they reduce game species	0.33	0.471	0	1
WTP	1 if the participant is willing to pay for the management of red foxes in Greece, when they carry diseases	0.811	0.392	0	1
Attitudes toward red foxes	How strongly the participant agrees of disagrees with attitude statements (1 = strongly disagree, 2 = disagree, 3 = neither, 4 = agree, 5 = strongly agree)	3.944	0.746	1	5
Knowledge about red foxes	How strongly the participant agrees of disagrees with knowledge statements (1 = strongly disagree, 2 = disagree, 3 = neither, 4 = agree, 5 = strongly agree)	3.976	0.615	1	5
Red fox likeability	How strongly the participant likes of dislikes red foxes (1 = strongly dislike, 2 = dislike, 3 = neither, 4 = like, 5 = strongly like)	4.022	0.943	1	5
Red fox fear	How safe or afraid would the participant feel if encountering red foxes (1 = very safe, 2 = safe, 3 = neither, 4 = afraid, 5 = very afraid)	1.862	1.152	1	5
Age	Years of age	45.84	17.37	18	82
Gender	1 if the participant is a woman	0.521	0.5	0	1
Educational level	1 if lower, 2 if higher	1.312	0.426	1	2
Income	Annual household income (€ × 1000)	19.943	14.842	0	150
Residence	1 if the participant lives in a rural area, 0 if the participant lives in an urban area	0.321	0.5	0	1
Occupation	1 if the participant is a farmer, 0 if the participant is not a farmer	0.324	0.454	0	1
Hunting	1 if the participant is a hunter, 0 if the participant is not a hunter	0.255	0.308	0	1
Pet ownership	1 if the participant owns a pet, 0 if the participant does not own a pet	0.587	0.493	0	1

In the second step, the interval model proposed by Welsh and Poe (1998) was used for analyzing data in a multiple-bounded payment card format, including data for the respondents who answered yes in the first, yes/no, model (i.e., the degree of certainty for paying each amount presented to them). We adopted the “probably yes” approach, such that ‘‘definitely yes’’ and ‘‘probably yes” mean ‘‘yes,’’ and ‘‘not sure,’’ ‘‘probably no,’’ and ‘‘definitely no’’ mean ‘‘no’’. This approach gives results similar to other standard discrete choice models, such as dichotomous choice, payment card, and open-ended (Hanemann et al. 1991; Welsh and Poe 1998). After recoding the multiple-bounded data into terms of ‘‘yes’’ and ‘‘no’’, it can be treated in the same way as ordinary double-bounded discrete choice data (Hanemann et al. 1991; Broberg and Brännlund 2008). Under the interval model approach, the highest bid the respondents accept and the lowest bid that they do not accept were considered. If *A*^*L*^ is the highest “yes” bid, and *A*^*U*^ the lowest “no” bid, the maximum WTP is *A*^*L*^ ≤ *WTP* < *A*^*U*^. Then, given a distribution function *F* for WTP, the likelihood is (Hanemann et al. 1991; Welsh and Poe 1998; Broberg and Brännlund 2008):

1 $$lnL= \sum\nolimits_{i=1}^{N}[{\text{ln}}(F({A}^{U}) - F\left({A}^{L}\right)]$$

And assuming a log-logistic distribution:

2 $$F\left({A}^{U}\right)={\left(1+ {e}^{\delta {\rm X}-\alpha {\text{ln}}\left({A}_{i}^{U}\right)}\right)}^{-1}$$

and

3 $$F\left({A}^{L}\right)={\left(1+ {e}^{\delta {\rm X}-\alpha {\text{ln}}\left({A}_{i}^{L}\right)}\right)}^{-1}$$

where *Χ* is the vector of covariates, and *δ* is the corresponding parameter vector. The parameter *α* corresponds to the bid and can be interpreted as the marginal utility of money. Mean WTP is then calculated as:

4 $${\rm M}WTP= {e}^{\frac{\delta {\rm X}}{\alpha }+ {(\frac{{\alpha }^{-1}}{2})}^{2}}$$

### Data analysis

We included in our models the independent variables with low variance inflation factors (VIF < 5) and correlations (*r*_*s*_ < 0.7). VIFs were calculated using the function *vifstep* of the *usdm* R package (Naimi et al. 2014) and correlations using the function *cor.test* of the *ggpubr* R package (Kassambara 2020). All VIFs were < 1.806 (Table S2) and correlations < 0.563 (Table S3), therefore all variables were included in the models.

The binary logistic regression, yes/no, model was fitted using the function *glm* (binomial distribution with logit link function) of the *stats* R package (R Core Team 2020). Odds ratios were calculated using the *logitor* and marginal effects at the means using the *logitmfx* functions of the *mfx* R package (Fernihough 2019). The second, highest yes/lowest no bids, model was fitted with a log-logistic distribution using the function *dbchoice* of the *DCchoice* R package (Nakatani et al. 2021). Confidence intervals (95% CI) for the mean WTP were calculated with the nonparametric bootstrap method using the *bootCI* function. All statistical analyses were performed in program R 4.0.2 (R Core Team 2020). The significance level was set at *α* = 0.05.

## Results

### Sociodemographics

We collected data from 746 respondents (response rate 91%) of which 51.2% were females, 31.2% had higher education, 32.4% were farmers, 32.1% were rural residents, and the age proportions were 30.3%, 32.8%, and 36.9% in the 18–34, 35–54, and 55 + -year-old age classes respectively (Table 1). The proportions recorded in this study were similar to the proportions recorded for the population of the regions of Western Macedonia, Central Macedonia, and Eastern Macedonia and Thrace (Table S4).

### Attitudes toward and knowledge about red foxes

Two significant factors were identified by factor analysis (eigenvalue ≥ 1), labeled as the “attitude” (eigenvalue 2.5) and “knowledge” (eigenvalue 1.5) factors that explained 44.8% of the variance (Table 2 (attitude: 27.9%; knowledge: 16.9%). The attitude factor included the five attitude statements (mean 3.944 ± 0.746 SD) and explained 27.9% of the variance. The great majority of the respondents stated that foxes are valuable to nature (93.9%), that they would like to see foxes in the wild (74.4%), and that management should aim at the increase of current fox populations (91.3%). Also, more than half of the respondents would like to coexist with foxes (56.4%), considering them valuable to people (51.1%). The knowledge factor included the four knowledge statements (3.976 ± 0.615) and explained the 16.9% of the variance. Most of the participants knew that foxes prey on domestic animals (94.2%), that foxes may carry rabies (73.4%), that foxes belong to the same family as dogs and wolves (68.3%), and that foxes are omnivores (59.0%). These identified factors were used as predictors in the WTP regression models.

**Table 2:** Results of principal components factor analysis of survey participants' (*n* = 746) attitudes toward and knowledge about red foxes. Descriptive statistics, factor loadings, factor eigenvalues, % variance explained, and factor reliability are given

Statements	Mean^b^	SD	Attitude	Knowledge
Red foxes must exist because they are valuable to nature	4.564	0.682	*0.676*	0.175
Red foxes must exist because they are valuable to people	3.253	1.321	*0.719*	–0.058
I would like to see red foxes in the wild	3.949	1.18	*0.721*	–0.157
I would like to coexist with red foxes	3.455	1.272	*0.644*	0.169
Management should aim at the increase of current red fox populations	4.5	0.83	*0.718*	–0.104
Red foxes may carry rabies	4.103	0.987	–0.244	*0.655*
Red foxes prey on domestic animals (e.g., poultry, pets)	4.513	0.722	0.066	*0.43*
Red foxes belong to the same family as dogs and wolves	3.747	1.107	0.139	*0.625*
Red foxes are omnivores	3.542	1.194	–0.045	*0.646*
Eigenvalue			2.508	1.52
% Variance explained			27.871	16.883
Cronbach's alpha			0.799	0.718

Factor loadings in italics indicate group membership; ^b^ Range: 1 (strongly disagree) – 5 (strongly agree)

### Willingness to pay for red fox impact management

#### Red foxes attack domestic animals

When asked for their WTP only for this program, 51.9% (*n* = 387) of the respondents stated that they were willing to pay for the management of foxes when they attack domestic animals. The proportion of correct predictions for the whole sample was 71.9% for the logistic regression model (Table 3). Positive attitudes toward foxes were associated with low WTP (*p* = 0.040), with the probability of WTP for managing their impacts decreasing by 9.9% per unit of increase in attitudes. Knowledge about foxes was positively associated with WTP (*p* = 0.004), with the probability of WTP increasing by 13.3% per unit of increase in knowledge. Fear of foxes was positively associated with WTP (*p* < 0.001), with the probability of WTP increasing by 8.7% per unit of increase in fear. WTP was higher for females than males (*p* = 0.004), with the probability of WTP being higher for females than males by 7.0%. High household income was associated with high WTP (*p* = 0.023), with the probability of WTP increasing by 2.7% per unit of increase in income. Being a rural resident (*p* < 0.001), a farmer (*p* = 0.002), and having pets (*p* = 0.003) increased the probability of WTP by 9.4%, 7.7%, and 7.2% respectively.

**Table 3:** Results of the binary logistic regression willingness to pay models (yes/no, *n* = 746)

Variable	Red foxes attack domestic animals			Red foxes reduce game			Red foxes carry disease		
	Odds ratio	Marginal effects	*p*	Odds ratio	Marginal effects	*p*	Odds ratio	Marginal effects	*p*
Attitude	0.673	–0.099	*0.040*	1.105	0.022	0.479	1.144	0.021	0.443
Knowledge	1.572	0.113	*0.004*	1.016	0.004	0.929	1.69	0.080	*0.006*
Likeability	1.044	0.011	0.712	0.771	–0.057	*0.038*	1.103	0.015	0.321
Fear	1.419	0.087	< *0.001*	1.283	0.055	*0.009*	1.558	0.068	< *0.001*
Age	0.995	–0.001	0.433	1.01	0.002	0.156	0.993	–0.001	0.434
Gender (Female)	1.326	0.07	*0.004*	0.597	–0.114	*0.009*	1.042	0.006	0.871
Education (Higher)	0.933	–0.017	0.742	0.44	–0.181	*0.001*	0.493	–0.108	*0.006*
Income	1.113	0.027	*0.023*	1.001	0.000	0.254	1.073	0.011	*0.042*
Residence (Rural)	1.456	0.094	< *0.001*	0.781	–0.055	0.186	0.86	–0.023	0.518
Occupation (Farmer)	1.364	0.077	*0.002*	0.903	–0.023	0.654	1.416	0.053	< *0.001*
Hunting (Hunter)	0.987	–0.003	0.964	1.526	0.093	< *0.001*	2.453	0.138	< *0.001*
Pet ownership (Yes)	1.332	0.072	*0.003*	0.789	0.052	0.172	1.127	0.018	0.591
Nagelkerke's *R*^2^	0.271			0.323			0.336		
− 2LogLik	842.551			749.425			551.023		
AIC_c_	869.048			775.922			577.52		

The interval regression model estimated a mean amount of WTP at €34.1 (95% CI: 26.6–41.6) (Table 4). Considering the mean value, confidence intervals, the proportion of the respondents who were willing to pay, and the number of households in the study area, an annual amount of €18.7 million (min €14.6 million, max €22.8 million) could be collected in taxes for the management of foxes when they attack domestic animals.

**Table 4:** Results of the interval regression willingness to pay models (highest yes/lowest no bids)

Variable	Red foxes attack domestic animals (*n* = 387)	Red foxes reduce game (*n* = 246)	Red foxes carry disease (*n* = 605)
	Estimate (SE)	Estimate (SE)	Estimate (SE)
Attitude	–0.310* (0.125)	–0.248* (0.114)	0.017 (0.108)
Knowledge	0.418** (0.144)	0.010 (0.128)	0.320** (0.127)
Likeability	–0.358** (0.136)	–0.269** (0.095)	–0.229** (0.088)
Fear	0.222** (0.082)	0.076 (0.065)	0.0007 (0.061)
Age	0.009 (0.006)	–0.024*** (0.005)	–0.124*** (0.025)
Gender (Female)	0.671*** (0.181)	–0.362* (0.158)	0.167 (0.148)
Education (Higher)	0.100 (0.235)	–0.156 (0.165)	–0.401** (0.164)
Income	0.007* (0.001)	0.008* (0.002)	0.009* (0.004)
Residence (Rural)	0.810*** (0.165)	0.095 (0.137)	–0.091 (0.128)
Occupation (Farmer)	0.523*** (0.142)	–0.093 (0.137)	0.073 (0.158)
Hunting (Hunter)	0.495*** (0.121)	0.545*** (0.236)	0.314* (0.132)
Pet ownership (Yes)	0.926*** (0.184)	0.022 (0.139)	0.321* (0.126)
Nagelkerke's *R*^2^	0.298	0.354	0.372
− 2LogLik	1223.026	866.407	2347.306
AIC_c_	1250.002	893.976	2373.922
Mean WTP (€)	34.093	44.947	72.087
SD	74.867	91.915	118.449
95% CI	(26.620, 41.566)	(33.428, 56.466)	(62.630, 81.544)

* p < 0.05, ** *p* < 0.01, *** *p* < 0.001

Respondents with more positive attitudes toward foxes (*p* = 0.012) and who liked foxes more (*p* = 0.005) were willing to pay a lower amount for the management of foxes in this situation than those respondents with less positive attitudes toward foxes and who liked foxes less (Table 4). Respondents who had more knowledge about foxes (*p* = 0.011) and feared them (*p* = 0.007) were willing to pay a higher amount for the management of foxes than those respondents who had less knowledge about foxes and did not fear them. Females (*p* < 0.001), high income recipients (*p* = 0.037), rural residents (*p* < 0.001), farmers (*p* < 0.001), hunters (*p* < 0.001), and pet owners (*p* < 0.001) were willing to pay a higher amount for the management of foxes than males, low income recipients, urban residents, non-farmers, non-hunters, and non-pet owners respectively.

#### Red foxes reduce game species populations

When asked for the WTP only for this program, 33.0% (*n* = 246) of the respondents were willing to pay for the management of foxes when they reduce populations of game species. The proportion of correct predictions for the whole sample was 77.4% for the logistic regression model (Table 3). The likeability of foxes was negatively associated with WTP (*p* = 0.038), with the probability of WTP for managing foxes decreasing by 5.7% per unit of increase in likeability. Fear of foxes was positively associated with WTP (*p* = 0.009), with the probability of WTP increasing by 5.5% per unit of increase in fear. Being a male (*p* = 0.009), a hunter (*p* < 0.001), and having lower education (*p* = 0.001) increased the probability of WTP by 11.4%, 9.3%, and 18.1% respectively.

The interval regression model estimated a mean amount of WTP at €44.9 (95% CI: 33.4–56.5) (Table 4). Considering the mean value, confidence intervals, the proportion of the respondents who were willing to pay, and the number of households in the study area, an annual amount of €15.7 million (min €11.6 million, max €19.7 million) could be collected in taxes for the management of foxes when they reduce game species populations.

Respondents with more positive attitudes toward foxes (*p* = 0.013) and who liked foxes more (*p* = 0.008) were willing to pay a lower amount for the management of foxes in this situation than those respondents with less positive attitudes toward foxes and who liked foxes less (Table 4). Respondents who were male (*p* = 0.022), hunters (*p* < 0.001), and high income recipients (*p* = 0.039) were willing to pay a higher amount for the management of foxes than those respondents who were female non-hunters, and low income recipients. The amount that respondents were willing to pay for the management of foxes decreased with their age (*p* < 0.001).

#### Red foxes carry deadly disease

When asked for their WTP only for this program, 81.1% (*n* = 605) of the respondents stated that they were willing to pay for the management of foxes when they carry disease. The proportion of correct predictions for the whole sample was 84.1% for the logistic regression model (Table 3). Knowledge about foxes was positively associated with WTP (*p* = 0.006), with the probability of WTP increasing by 8.0% per unit of increase in knowledge. Fear of foxes was positively associated with WTP (*p* < 0.001), with the probability of WTP increasing by 6.8% per unit of increase in fear. High household income was associated with high WTP (*p* = 0.042), with the probability of WTP increasing by 1.1% per unit of increase in income. Being a farmer (*p* < 0.001), a hunter (*p* < 0.001), and having lower education (*p* = 0.006) increased the probability of WTP by 5.3%, 13.8%, and 10.8% respectively.

The interval regression model estimated a mean amount of WTP at €72.1 (95% CI: 62.6–81.5) (Table 4). Considering the mean value, confidence intervals, the proportion of the respondents who were willing to pay, and the number of households in the study area, an annual amount of €61.7 million (min €53.6 million, max €69.8 million) could be collected in taxes for the management of foxes when they carry disease.

Respondents with more knowledge about foxes (*p* = 0.012) and those who liked foxes less (*p* = 0.009) were willing to pay a higher amount for the management of foxes in this situation than those respondents with less knowledge about foxes and those who liked foxes more (Table 4). Respondents who were hunters (*p* = 0.012), pet owners (*p* = 0.011), had lower education (*p* = 0.007), and high income recipients (*p* = 0.027) were willing to pay a higher amount for the management of foxes than those respondents who were non-hunters, non-pet owners, had higher education, and were low income recipients. The amount that respondents were willing to pay for the management of foxes decreased with their age (*p* < 0.001).

## Discussion

### WTP for fox impact management

Our assessment of WTP for fox impact management under different situations showed that a great majority of respondents were willing to pay for managing foxes when they carry diseases. But only half of the respondents and one third of the respondents were willing to pay for managing foxes when they attack domestic animals and reduce game animals respectively. Also, the mean WTP was considerably higher for managing sick foxes than for managing impacts on domestic animals or game populations. Our previous report and analysis of public preferences for fox management strategies also showed that the public was more supportive of management when foxes carried rabies than the other, less severe, impact situations (as assessed by the “do nothing” management option; Liordos et al. 2020a). Previous similar studies for other wildlife species have also typically reported that people are more supportive of wildlife impact management for more severe (e.g., human and animal health and safety) than for less severe impacts (e.g., economic damage, aesthetic deterioration, and nuisance), in Greece (e.g., Liordos et al. 2017b; Kontsiotis et al. 2020) and other areas (e.g., West and Parkhurst 2002; Jacobs et al. 2014a, b; Sponarski et al. 2015; Doney et al. 2018).

Our study shows that a considerable amount of money can be collected from the public tax for fox management and mitigate their impacts. Although estimates of the cost of fox management are not currently available, considering the frequency and severity of fox impact incidents, we argue that the benefit/cost ratio of fox management is likely large and that a new tax would be sufficient to fund required actions and strategies for addressing any of the three fox impact situations. Apart from funding management strategies, funds should also be allocated to outreach actions for informing the public about the situation, the need for its management, and the efficiency of each proposed management strategy. The dissemination of such information will increase public support for fox management, especially of the most affected groups in each situation, such as farmers, hunters, and rural residents, whose contribution is critical for the successful implementation of management programs.

Our estimated rates of acceptance of payment and mean WTP fell within the ranges reported by previous studies from different areas. These studies have reported rates of acceptance of payment between 40 and 95% and mean WTP between €7.9 and €148.9 for the management of negative impacts of various wildlife taxa (Kerr and Cullen 1995; Bowker et al. 2003; van Eden et al. 2016; Mhuriro-Mashapa et al. 2017; Martínez-Jauregui et al. 2020; Table 5). Higher WTP values than those estimated in our study have been reported for the most damaging and potentially dangerous large African mammals, such as the African elephant, African buffalo, African lion, and spotted hyena (van Eden et al. 2016; Mhuriro-Mashapa et al. 2017) and invasive alien species, such as the brush-tailed possum in New Zealand (Kerr and Cullen 1995). The WTP for managing foxes estimated in our study was comparable to that estimated for the red deer that caused severe economic losses to farmers in Spain and the U.S.A. (Bowker et al. 2003; Martínez-Jauregui et al. 2020; Table 5). Rabies incidents, although lethal, are very rare in Greece (Tsiodras et al. 2013; Tasioudi et al. 2014). Also, impacts on agriculture and game are usually medium to low, although frequent (Papageorgiou et al. 1988; Vlachos et al. 2006; Liordos et al. 2020a). Considering these factors, we can argue that there is a high interest in fox management among the Greek public, for all three impact situations, as compared to the interest in the management of other wildlife taxa, especially the red deer.

**Table 5:** WTP for managing the impacts of various wildlife taxa. Mean WTP and GDP per capita values correspond to the study year and are given in 2015 € values

Taxon	Impact situation	Mean WTP (€)	GDP per capita (€)^a^	WTP/GDP	Year of study	Country	Source
African elephant (*Loxodonta africana*) African buffalo (*Syncerus caffer*) African lion (*Panthera leo*) Spotted hyena (*Crocuta crocuta*)	Crop and livestock damage	148.9^b^	1445.1	0.1030	2015	Zimbabwe	Mhuriro-Mashapa et al. (2017)
Brush-tailed possum (*Trichosurus vulpecula*)	Forest vegetation damage	262.0^c^	26,387.3	0.0099	1993	New Zealand	Kerr and Cullen (1995)
Brush-tailed possum (*Trichosurus vulpecula*)	Forest vegetation damage	180.1^c^	26,387.3	0.0068	1993	New Zealand	Kerr and Cullen (1995)
African elephant (*Loxodonta africana*)	Crop damage	7.9^d^	1462.2	0.0054	2014	Kenya	van Eden et al. (2016)
Red deer (*Cervus elaphus*)	Livestock predation	137.0	30,364.6	0.0045	2018	Spain	Martínez-Jauregui et al. (2020)
Red fox (*Vulpes vulpes*)	Carry disease	72.1	18,582.1	0.0039	2017	Greece	Here
Red deer (*Cervus elaphus*)	Damage to wildlife	80.0	30,364.6	0.0026	2018	Spain	Martínez-Jauregui et al. (2020)
Red deer (*Cervus elaphus*)	Damage to vegetation	75.0	30,364.6	0.0025	2018	Spain	Martínez-Jauregui et al. (2020)
Red fox (*Vulpes vulpes*)	Reduce game	45.0	18,582.1	0.0024	2017	Greece	Here
Red deer (*Cervus elaphus*)	Urban property damage	66.1^e^	29,967.7	0.0022	1996	U.S.A	Bowker et al. (2003)
Red fox (*Vulpes vulpes*)	Attack domestic animals	34.1	18,582.1	0.0018	2017	Greece	Here
Red deer (*Cervus elaphus*)	Urban property damage	49.8^f^	29,967.7	0.0017	1996	U.S.A	Bowker et al. (2003)
Asian elephant (*Elephas maximus*)	Damage to crops and property, human casualties	1.24	720.7	0.0017	2012	Nepal	Neupane et al. (2017)
African elephant (*Loxodonta africana*)	Crop damage	2.3^ g^	1462.2	0.0016	2014	Kenya	van Eden et al. (2016)

^a ^Source: Country search in World Bank web site (World Bank 2022); ^b^ Farmers; ^c^ Different models; ^d^ Pastoralists; ^e^ Lethal control; ^f^ Non-lethal control; ^g^ Non-pastoralists

### WTP, attitudes, knowledge, and emotions

Survey respondents had positive attitudes toward foxes, with those being more positive being less willing to pay (in the foxes reduce game situation) and also willing to pay a lower amount than those being more negative (in the foxes attack domestic animals and reduce game situations). Previous studies have shown that people with positive attitudes toward wildlife and nature, in general, are more supportive of species conservation than people with negative attitudes (e.g., Knight 2008; Liordos et al. 2017a; Fonseca et al. 2021; Kontsiotis et al. 2022). Based on these findings and considering that studies about the effects of attitudes on the WTP for wildlife impact management were not available, we argue that people who are supportive of wildlife conservation will also generally be more supportive of their welfare and will not easily accept the control of wildlife populations. On the other hand, previous studies have shown that the public generally accepts the management, even lethal strategies, of wildlife in situations where the health and life of people are at risk. In northern Greece, the public accepted the lethal management of foxes when they transfer rabies but not when they attack livestock or game (Liordos et al. 2020a), the lethal management of wild boars, European badgers (M*eles meles*), and coypus (*Myocastor coypu*) when they transfer disease but not when they damage crops (Liordos et al. 2017b; Kontsiotis et al. 2020). The absence of effects of attitudes on the WTP for managing foxes when they carry disease might partly be explained by the priority that people give to the value of human life during life-threatening situations.

Survey respondents with more knowledge about the biology and ecology of foxes were more willing to pay and were willing to pay a higher amount for the management of foxes (except in the foxes reduce game situation) compared to others with less knowledge about this species. In general, other studies have also shown that people with high knowledge about wildlife were more supportive of the conservation of endangered species (Knight 2008; Liordos et al. 2017a, 2018, 2019) and the management of wildlife impacts (Mhuriro-Mashapa et al. 2017). Mhuriro-Mashapa et al. (2017) found that the knowledge of management issues was a significant positive predictor of WTP for human-wildlife conflict management. In our study, respondents were aware of foxes’ predation on domestic animals and that they are vectors of rabies. However, we did not ask respondents about their knowledge of possible predation on game. The absence of effects of knowledge on the WTP for managing foxes when they reduce game might be explained by a lack of knowledge of the foxes’ feeding habits, an issue for future research.

The great majority of the respondents considered foxes as highly likeable animals and did not express fear of them. Mammals, such as the fox, are among the most likeable wildlife species due to their phylogenetic resemblance to humans (Metrick and Weitzman 1998; Tisdell et al. 2006) and their positive portrayal by societies (Czech et al. 1998). Likeability was negatively and fear positively associated with WTP for fox impact management. Previous studies have also reported that people who like a certain wildlife species more and feared them less were more supportive of their conservation and were less willing to pay for the management of their impacts in most situations (Knight 2008; Johansson et al. 2012; Liordos et al. 2017a, 2021; Fonseca et al. 2021; Kontsiotis et al. 2022). However, fear of the animal did not affect WTP for fox management when they reduce game or carry disease. This might happen because only hunters, among the respondents, were interested in fox management when they reduce game. Also, people generally accept management, irrespective of group membership, when wildlife species can transfer disease to humans and their animals (Liordos et al. 2017b; Kontsiotis et al. 2020). These findings suggested that fear of disease and not fear of the animal itself was the decisive factor.

### WTP and sociodemographics

Females were more willing to pay when foxes attack domestic animals, while males were more willing to pay when foxes reduce game. WTP when foxes reduce game or carry disease decreased with age, while respondents who had a lower level of education were more willing to pay for managing sick foxes. Kerr and Cullen (1995) found that females and older people were more willing to pay than males and younger people to protect vulnerable species from brush-tailed possum infestation in New Zealand. Bowker et al. (2003) reported that females were willing to pay more than males for non-lethal control and less than males for lethal control of red deer to reduce damage to suburban properties in the United States. Educational level was positively related to WTP for reducing damage to crops, property, and livestock and human casualties from Asian elephants in Nepal (Neupane et al. 2017) and from African elephants, African buffalos, African lions, and spotted hyenas in Zimbabwe (Mhuriro-Mashapa et al. 2017). Females learn early in their lives to become responsible to others, nonaggressive, caring, and compassionate, regarding the natural world with empathy and care (“The Ethic of Care”; Gellar 1995). Therefore, females usually show more affection toward animals, more intolerance of animal cruelty and exploitation, and have more pro-conservation attitudes than males (Knight 2008; Teel and Manfredo 2010; Liordos et al. 2018). The higher WTP of females than males when foxes attack domestic animals might be due to the precedence of care for their children over care for wildlife in the proximity of their homes, as also observed by Kerr and Cullen (1995). Males, characterized by fairness, logic, hierarchy, assertiveness, and competition (Gellar 1995), were more willing to pay for the protection of game and consequently the practice of hunting, an activity in which females rarely engage in Greece (Raftogianni et al. 2022). Neupane et al. (2017) and Mhuriro-Mashapa et al. (2017) contended that educated and more experienced older people have better access to information and can better understand the importance and scope of wildlife conservation and impact management issues. Our results suggested that there might existed an interplay between the conservation and management of foxes, resulting in precedence of the former over the latter in educated and older respondents. However, further research is needed to better understand the effects of age and educational level on WTP for fox impact management.

WTP for fox impact management increased with household income in all the situations. Income has most often been positively associated with WTP for wildlife conservation and impact management, but not always. In Greece, WTP increased with income for the conservation of Balkan chamois (*Rupicapra rupicapra balcanica*) (Liordos et al. 2023), northern white-breasted hedgehogs (*Erinaceus roumanicus*) (Kontsiotis et al. 2023), and snakes (Liordos et al. 2024) but income did not affect WTP for the conservation of bats (Liordos et al. 2021). In other countries, WTP also increased with income for managing damage caused by African elephants, African buffalos, African lions, and spotted hyenas in Zimbabwe (Mhuriro-Mashapa et al. 2017) and Asian elephants in Nepal (Neupane et al. 2017) and for the conservation of grey wolves (*Canis lupus*) in the U.S.A. (van Eeden et al. 2021) and giant pandas (*Ailuropoda melanoleuca*) in China (Ma et al. 2016). However, income did not affect WTP for managing damage caused by brush-tailed possums in New Zealand (Kerr and Cullen 1995) and for the conservation of Bengal tigers (*Panthera tigris tigris*) in Nepal (Bhattarai et al. 2021) and Mauritian flying foxes (*Pteropus niger*) in Mauritius (Jaunky et al. 2021). According to economic theory, fox impact management represented a “normal good”, where WTP would increase with income, for the Greek residents (Haque 2005).

Rural residents and farmers were more interested in protecting domestic animals from predation, while hunters were willing to pay for fox impact management in all situations. Previous studies have also reported that farmers strongly supported management strategies for minimizing wildlife impacts on their livelihoods, while hunters supported strategies that do not negatively affect their favorite game (Frank et al. 2015; Kontsiotis et al. 2020; Liordos et al. 2020a). Kontsiotis et al. (2020) studied public preferences for management strategies when wild boars and Eurasian badgers raid crops or transfer disease in Greece. Farmers were strongly supporting management strategies in all situations, while hunters were more reluctant to accept the reduction in numbers of a game species (i.e., the wild boar) than of a non-game species (i.e., the Eurasian badger). These findings seem to explain why farmers were more willing to pay when foxes attacked domestic animals, while hunters were more willing to pay when foxes reduced game. Rural residents share the same environment with foxes; therefore, they are more exposed to negative impacts compared to urban residents (Karlsson and Sjöström 2007). This might explain their higher WTP for reducing fox impact on domestic animals.

Pet owners were more willing to pay for managing fox predation on domestic animals and sick foxes. Pet owners have an interest in all animals, are concerned about their welfare, and usually support wildlife conservation (Shuttlewood et al. 2016; Liordos et al. 2020b). In our case, it seemed that pet owners were more worried about the protection of their beloved pets from fox predation and transmission of disease, with the latter life-threatening situation being also important for themselves.

### Study limitations

The sample’s proportions of age, gender, educational level, place of residence, and occupation were similar to the proportions recorded by ELSTAT (2011) for the sampled population, therefore this was a representative sample. Regional data on income, hunting membership, and pet ownership were not available for comparison.

We tried to address biases associated with WTP surveys (Greene 2018). Social desirability bias, the possibility that respondents might state what they believe is socially acceptable, rather than their true opinions, was reduced by securing anonymity at all stages of the survey and allowing participants to self-complete the questionnaire (Vaske 2019). We induced framing bias (the way the WTP question is framed) because we wanted participants to be informed about fox impact situations and their importance. Hypothetical bias, that is people state what they would pay for a good or service, rather than what they actually pay, overestimates WTP by 21% on average (Schmidt and Bijmolt 2020).

## Conclusions

We assessed the social value of fox impact management in three different situations by using a CVM. More respondents were willing to pay and were willing to pay a higher amount when foxes transfer disease, a situation that could potentially affect more people and threaten their lives, than for situations that could mostly affect certain stakeholder groups, such as farmers when foxes attack domestic animals and hunters when foxes reduce game. Attitudes toward foxes, knowledge about the biology and ecology of foxes, emotions toward foxes, and sociodemographic characteristics variously affected WTP in different situations. Future research should examine the public’s knowledge about the feeding habits of foxes and carry out a deeper analysis of the effects of age, gender, and educational level on WTP for fox impact management. Also, the perceptions of fox management of other stakeholder groups, such as officials of governmental bodies (e.g., Forest Service, Veterinary Service, Ministry of Energy and the Environment) and environmental nongovernmental organizations should be investigated.

When projecting the mean WTP to the target population, considerable funds could be collected for managing any of the impact situations. These funds and further research could be used (a) for designing, implementing, and assessing education and outreach programs aimed at informing the public, especially people not willing to pay for fox management such as those with negative attitudes toward foxes, low knowledge about the biology and ecology of foxes, who did not like foxes, and various sociodemographic groups that behaved differently depending on the management situation, and also key stakeholder groups, such as farmers and hunters, on the necessity of management and the most efficient management strategies, and (b) for implementing suitable impact management programs through the integration of the variable effects of cognitions, emotions, and different sociodemographic groups. The successful implementation of such programs would not be possible without the consent, and in some instances the contribution, of the public. Our findings will be valuable for advising the management process.

### Supplementary Information

Below is the link to the electronic supplementary material.

Supplementary file1 (PDF 193 KB)

## Data Availability

The datasets used in the study are available from the corresponding author on reasonable request.

## Code availability

The code used in the study is available from the corresponding author on reasonable request.
