# Supplementary material for: Economic valuation of a mesocarnivore’s impact management
Source: Environ Sci Pollut Res Int. 2024 Apr 22;31(22):32111–25. doi: 10.1007/s11356-024-33398-4 (PMC11133192; doi:10.1007/s11356-024-33398-4)
Supplement: Supplementary file 1 — Supplementary file1 (PDF 193 KB) [file 11356_2024_33398_MOESM1_ESM.pdf]

Economic valuation of a mesocarnivore’s impact management

Vasileios J. Kontsiotis · Foteini Emmanouilidou · Vasilios Liordos
Department of Forest and Natural Environment Sciences
International Hellenic University, P.O. Box 172, 66100 Drama, Greece
Correspondence: liordos@for.ihu.gr

APPENDIX A
SUPPLEMENTARY FILES

Table S1 The survey instrument

A. WILLINGNESS TO PAY

Below are offered three red fox impact situations.
ATTENTION! You are asked to rate your willingness to pay for the implementation of each situation, treating it as independent of the others, meaning that you would be asked eventually to pay for one program only.

A1.1. WTP – Red foxes attack domestic animals
When there is evidence that red foxes attack domestic animals, such as lambs, poultry, and pets, causing considerable economic damage, would you support a governmental management program to reduce red fox impact by paying an amount annually for the next five years?
(please circle one answer)

YES
NO

If the answer is YES, please proceed to the next question. If the answer is NO, please proceed to the next impact situation.

A1.2. PAYMENT CARD FORMAT
Below are listed several amounts of an annual tax that you will have to pay for the next five years for reducing red fox impact. Check for each amount how certain you are about paying that amount:
(please circle one answer for each amount)

€ 1	€ 5	€ 10	€ 20	€ 40
Definitely yes	Definitely yes	Definitely yes	Definitely yes	Definitely yes
Probably yes	Probably yes	Probably yes	Probably yes	Probably yes
Not sure	Not sure	Not sure	Not sure	Not sure
Probably no	Probably no	Probably no	Probably no	Probably no
Definitely no	Definitely no	Definitely no	Definitely no	Definitely no

€ 80	€ 150	€ 300	€ 500
Definitely yes	Definitely yes	Definitely yes	Definitely yes
Probably yes	Probably yes	Probably yes	Probably yes
Not sure	Not sure	Not sure	Not sure
Probably no	Probably no	Probably no	Probably no
Definitely no	Definitely no	Definitely no	Definitely no

A2.1. WTP – Red foxes reduce game populations

When there is evidence that red foxes attack game species, such as hare and partridges, and reduce their populations, would you support a governmental management program to reduce red fox impact by paying an amount annually for the next five years?
(please circle one answer)

YES
NO

If the answer is YES, please proceed to the next question. If the answer is NO, please proceed to the next impact situation.

A2.2. PAYMENT CARD FORMAT

Below are listed several amounts of an annual tax that you will have to pay for the next five years for reducing red fox impact. Check for each amount how certain you are about paying that amount:
(please circle one answer for each amount)

€ 1	€ 5	€ 10	€ 20	€ 40
Definitely yes	Definitely yes	Definitely yes	Definitely yes	Definitely yes
Probably yes	Probably yes	Probably yes	Probably yes	Probably yes
Not sure	Not sure	Not sure	Not sure	Not sure
Probably no	Probably no	Probably no	Probably no	Probably no
Definitely no	Definitely no	Definitely no	Definitely no	Definitely no

€ 80	€ 150	€ 300	€ 500
Definitely yes	Definitely yes	Definitely yes	Definitely yes
Probably yes	Probably yes	Probably yes	Probably yes
Not sure	Not sure	Not sure	Not sure
Probably no	Probably no	Probably no	Probably no
Definitely no	Definitely no	Definitely no	Definitely no

A3.1. WTP – Red foxes carry deadly disease

When there is evidence that red foxes attack are vectors of deadly diseases, such as rabies, and threaten the lives of people and their animals, would you support a governmental management program to reduce red fox impact by paying an amount annually for the next five years?
(please circle one answer)

YES
NO

If the answer is YES, please proceed to the next question. If the answer is NO, please proceed to Section B1.

A3.2. PAYMENT CARD FORMAT

Below are listed several amounts of an annual tax that you will have to pay for the next five years for reducing red fox impact. Check for each amount how certain you are about paying that amount:
(please circle one answer for each amount)

€ 1	€ 5	€ 10	€ 20	€ 40
Definitely yes	Definitely yes	Definitely yes	Definitely yes	Definitely yes
Probably yes	Probably yes	Probably yes	Probably yes	Probably yes
Not sure	Not sure	Not sure	Not sure	Not sure
Probably no	Probably no	Probably no	Probably no	Probably no
Definitely no	Definitely no	Definitely no	Definitely no	Definitely no

€ 80	€ 150	€ 300	€ 500
Definitely yes	Definitely yes	Definitely yes	Definitely yes
Probably yes	Probably yes	Probably yes	Probably yes
Not sure	Not sure	Not sure	Not sure
Probably no	Probably no	Probably no	Probably no
Definitely no	Definitely no	Definitely no	Definitely no

B1. ATTITUDES AND KNOWLEDGE

Please select a box for indicating how strongly do you agree with each of the following statements.

Statements	Strongly disagree	Disagree	Neither	Agree	Strongly agree
Red foxes must exist because they are valuable to nature.					
Red foxes must exist because they are valuable to people.					
I would like to see red foxes in the wild.					
I would like to coexist with red foxes.					
Management should aim at the increase of current red fox populations.					
Red foxes may carry rabies.					
Red foxes prey on domestic animals (e.g., poultry, pets).					
Red foxes belong to the same family as dogs and wolves.					
Red foxes are omnivores.					

B2. EMOTIONS

How strongly attractive or unattractive do you red foxes? (please check appropriate box)

	Strongly unattractive	Unattractive	Neither	Attractive	Strongly attractive
I find foxes					

How safe or afraid would you feel if encountered red foxes? (please check appropriate box)

	Very safe	Safe	Neither	Afraid	Very afraid
I would feel around snakes					

C. SOCIODEMOGRAPHICS

What is your gender? (please check appropriate box)

Female ☐
Male ☐
Non-binary ☐

How old are you? ☐

What is your annual household income?

____

What is your occupation? (please check appropriate box)

Farmer ☐
Other ☐

What is your educational level? (please check appropriate box)

Lower (primary and secondary) ☐
Higher (post-secondary) ☐

Do you live in an urban or in a rural area? (please check appropriate box)

Rural area ☐
Urban area ☐

Are you a licensed hunter? (please check appropriate box)

Yes ☐
No ☐

Do you own a pet? (please check appropriate box)

Yes ☐
No ☐

Table S2 Variance inflation factors (VIFs) of variables used in willingness to pay models

Knowledge	1.658
Likeability	1.139
Fear	1.806
Age	1.578
Gender (Female)	1.225
Education (Higher)	1.474
Income	1.192
Residence (Rural)	1.115
Occupation (Farmer)	1.142
Hunting (Hunter)	1.250
Pet ownership (Yes)	1.099

Table S3 Spearman rank correlations (r_s) of variables used in willingness to pay models

	Attitude	Knowledge	Likeability	Fear	Age	Gender (Female)	Education (Higher)	Income	Residence (Rural)	Occupation (Farmer)	Hunting (Hunter)
Knowledge	0.014										
Likeability	0.563	0.016									
Fear	-0.326	-0.022	-0.219								
Age	-0.029	0.274	-0.065	-0.038							
Gender (Female)	-0.032	-0.093	0.131	0.475	-0.158						
Education (Higher)	0.207	0.059	0.253	-0.075	-0.159	0.036					
Income	0.19	0.023	0.194	-0.088	0.232	-0.042	0.123				
Residence (Rural)	-0.197	0.067	-0.25	0.058	0.021	0.008	-0.199	-0.127			
Occupation (Farmer)	-0.093	0.178	-0.177	-0.132	0.105	-0.211	-0.078	0.084	0.057		
Hunting (Hunter)	-0.081	0.16	-0.215	-0.179	0.142	-0.304	-0.029	0.016	0.021	0.145	
Pet ownership (Yes)	0.016	0.056	-0.02	-0.141	-0.009	-0.057	-0.024	0.001	0.188	0.112	0.204

Table S4 Comparison of sociodemographic characteristics (%) between the sample (n = 746) and the study area (n = 2,774,000; ELLSTAT 2011)

	Sample	Study population	χ^2	p
Gender				
Female	52.1	51.2	0.243	0.569
Male	47.9	48.8		
Age				
18-34	30.3	28.4		
35-54	32.8	36.2	4.591	0.101
55+	36.9	34.4		
Educational level				
Higher	31.2	26.7	1.245	0.247
Lower	68.8	73.3		
Occupation				
Farmers	32.4	29.3	3.292	0.064
Non-farmers	67.6	70.7		
Place of residence				
Rural	32.1	28.9	3.529	0.055
Urban	67.9	71.1		
